# Odontalgia

**Published:** 1859-10

**Authors:** J. P. H. Brown

**Affiliations:** Dentist, Atlanta, Geo.


### ARTICLE II.

*Odontalgia.* By J. P. H. BROWN, Dentist, Atlanta, Ga.

This "hell o' a' disease," as Burns laconically terms it, cannot with strict propriety be called a disease, but is always symptomatic of some organic or functional derangement, either of the organ in which the pain is located or of some other portion of the system. The pain in toothache is so variable that it is nearly impossible to give a correct idea of the sensations—*to feel* is the sure way *to know.* It may be so slight as to cause very little uneasiness; at other times it may be acute, sharp, lancinating, and throbbing; then, again, it may be dull, deep-seated and continuous, giving rise to the most intense agony. Sometimes it is erratic, and is confined to no particular tooth, now in one, then in another.

1. *Tooth-Ache from Exposure of the Nerve.*—When caries of a tooth has progressed so far as to lay bare the nerve filaments ramifying through the dental structure, acute pain is often felt in the tooth when any acid or sweet substance comes in contact with the carious surface. But when the decay has extended to the dental pulp, and it thus becomes exposed to the pressure and contact of foreign substances, such as particles of food, acrid secretions, &c., the pain often comes on with the suddenness of an electric flash, though it is seldom of long duration, until inflammation is set up in the living membrane and pulp-tissues. The pain now becomes boring, throbbing and lancinating. The capillaries of the pulp are engorged and distended, and the surrounding parts being solid and unyielding, the nerve, as a consequence, is subjected to pressure; hence the intense pain. The *throbbing* results from the pulsation of these vessels at each injection of the arteries, supplying them with blood.

*Treatment.*—When there is exposure of nerve, and only slight inflammation, there is a chance to save the tooth by destroying and removing the nerve, and then thoroughly filling the nerve cavity, and also the cavity of decay with gold. This department belongs exclusively to the dentist; but the physician is often called upon to "put something in" (to use common parlance) to relieve the pain. Many would prefer to do this to extraction. In my own practice I have found the following mixture to be of great benefit in giving temporary relief:

| | |
|---|---|
| ℞.—Spirit Ammon......℥ ss. | Ole. Caryophyl..........℥ii. |
| Chloroform..........℥i. | Tinc. Camph..............℥ij. |

*Misce.*

After cleaning out and drying the cavity, a small pellet of raw cotton should be moistened with this, and applied to the exposed pulp, repeating the application as occasion requires. Cold water or ice applied to the tooth will sometimes give relief. When the inflammation becomes deep seated no great benefit can be derived from the above treatment, and extraction should be recommended.

2. *Periodontitis.*—This form of tooth-ache arises from inflammation of the alveolo-dental periosteum. The symptoms in this variety differ somewhat from those characterizing the former. The pain is dull, deep-seated, and continuous. The tooth seems to rise from its socket, and to get longer than the rest. It feels sore to the touch, and when percussion is applied, pain is excited. The gums around the tooth become red and tumefied; the part of the face opposite the diseased organ often swells, indicating the formation of alveolar abscess.

This variety of odontalgia is often caused by filling over inflamed or dead nerves; but more frequently by the confinement of pus in the internal cavity, by cold, or by mechanical violence.

*Treatment.*—If the inflammation is taken in the first stage it may sometimes be successfully combatted by lancing the gums about the tooth, or by applying leeches to them, and then promoting the bleeding by holding warm water in the mouth. Hot pediluvia are not to be neglected. If the bowels are constipated, saline cathartics should be administered. The tooth should be extracted when there is a likelihood of the formation of alveola abscess. When abscess is forming, hot fomentations should be applied to the gums to promote the escape of the matter into the mouth. The practice of applying them to the face can not be too severely reprimanded, for they often cause the pus to escape, externally, through the cheek, hereby disfiguring the countenance and producing an unsightly scar.

3. *Sympathetic Tooth-ache* results from the transfer of nervous irritation, produced by functional or structural disturbance in some other portion of the body. The pain in this variety is generally erratic in its nature. It is confined to no particular tooth—attacking one, then another, and often assuming the neuralgic form. Sound teeth are quite as apt to become affected as decayed ones.

This form of odontalgia is frequently caused by pregnancy and uterine derangement, by indigestion, constipation, and exposure to malaria. When caused by the latter its attacks are always periodic. It often takes place during the existence of a rheumatic or arthritic diathesis. Dr. Harris relates a case of tooth-ache associated with gout of some fifteen years standing. "The paroxysms occurred at intervals of from three to six months, and from ten to twelve days before each attack, he (the patient) suffered from pain in the first right superior molar tooth, which was slightly affected with caries in the center of the grinding surface, but it had not yet penetrated to the pulp-cavity. The pain at first was not severe, but it gradually increased in intensity, and assumed a peculiar boring, and at times, grinding and lancinating character. His attacks of gout were confined to the joint of the big toe of his right foot, and as soon as the pain commenced here it subsided in his tooth. But when the paroysm of gout began to pass off, it again commenced in the tooth, where it continued for about two weeks."

In the *treatment* of sympathetic tooth-ache, local applications afford very little relief. The general state of the system must be attended to by an application of such means as are readily suggested to the mind of the intelligent physician. Extraction seems only to relieve the pain in a tooth to transplant in another. Sound teeth are thus often recklessly sacrificed to folly and ignorance, in order to gratify the whims of a pregnant, dyspeptic, gouty, or rheumatic patient, without affording one particle of benefit.

4. *Odontalgia from Exostosies.*—This variety is extremely difficult to diagnose from some of the other forms. A sound tooth is as liable to exostosies as a decayed one.

When ossific matter is first depositing upon the fang of a tooth, very little pain or uneasiness is experienced by the patient. A grumbling and pricking sensation may be all that is felt for a long time. But as the deposit progresses, the pain becomes more acute and lancinating, giving rise to the greatest agony. Those patients possessing a scorbutic diathesis, or a syphilitic taint, seem to be more particularly disposed to this affection.

The *treatment* is extraction.

